# Percent error of ultrasound examination to estimate fetal weight at term in different categories of birth weight with focus on maternal diabetes and obesity

**DOI:** 10.1186/s12884-022-04519-z

**Published:** 2022-03-23

**Authors:** Luisa Dittkrist, Julia Vetterlein, Wolfgang Henrich, Babett Ramsauer, Dietmar Schlembach, Michael Abou-Dakn, Ulrich Gembruch, Ralf L. Schild, Antonia Duewal, Ute M. Schaefer-Graf

**Affiliations:** 1 Department for Obstetrics, Medical Faculty, Humboldt University, Campus Rudolf-Virchow, Charité Berlin, Germany; 2 Department for Obstetrics and Gynaecology, St. Joseph Hospital, Berlin, Germany; 3 Clinic of Obstetric Medicine, Clinicum Vivantes Neukoelln, Berlin, Germany; 4 Department of Obstetrics and Prenatal Medicine, University of Bonn, Bonn, Germany; 5 Department of Obstetrics and Prenatal Medicine, DIAKOVERE Hannover, Hannover, Germany

**Keywords:** Estimated Fetal Weight, Percent Error, Birth Weight, Accuracy, Diabetes

## Abstract

**Background:**

Sonography based estimate of fetal weight is a considerable issue for delivery planning. The study evaluated the influence of diabetes, obesity, excess weight gain, fetal and neonatal anthropometrics on accuracy of estimated fetal weight with respect to the extent of the percent error of estimated fetal weight to birth weight for different categories.

**Methods:**

Multicenter retrospective analysis from 11,049 term deliveries and fetal ultrasound biometry performed within 14 days to delivery. Estimated fetal weight was calculated by Hadlock IV. Percent error from birth weight was determined for categories in 250 g increments between 2500 g and 4500 g. Estimated fetal weight accuracy was categorized as accurate ≤ 10% of birth weight, under- and overestimated by >  ± 10% – ± 20% and > 20%.

**Results:**

Diabetes was diagnosed in 12.5%, obesity in 12.6% and weight gain exceeding IOM recommendation in 49.1% of the women. The percentage of accurate estimated fetal weight was not significantly different in the presence of maternal diabetes (70.0% vs. 71.8%,* p* = 0.17), obesity (69.6% vs. 71.9%, *p* = 0.08) or excess weight gain (71.2% vs. 72%, *p* = 0.352) but of preexisting diabetes (61.1% vs. 71.7%; *p* = 0.007) that was associated with the highest macrosomia rate (26.9%). Mean percent error of estimated fetal weight from birth weight was 2.39% ± 9.13%. The extent of percent error varied with birth weight with the lowest numbers for 3000 g–3249 g and increasing with the extent of birth weight variation: 5% ± 11% overestimation in the lowest and 12% ± 8% underestimation in the highest ranges.

**Conclusion:**

Diabetes, obesity and excess weight gain are not necessarily confounders of estimated fetal weight accuracy. Percent error of estimated fetal weight is closely related to birth weight with clinically relevant over- and underestimation at both extremes. This work provides detailed data regarding the extent of percent error for different birth weight categories and may therefore improve delivery planning.

## Introduction

Ultrasonography (US) is an essential diagnostic tool for monitoring fetal growth during pregnancy.

Estimation of fetal weight (EFW) obtained near term is included in the counselling and planning of the delivery mode. EFW often provides guidance on the decision for or against an attempt of vaginal delivery or early induction in fetus with birth weight (BW) at the high or low end.

The increasing incidence of diabetes (DM) and obesity (OBS) worldwide is a challenge during pregnancy because both are correlated with fetal macrosomia. High BW is considered a major risk factor for shoulder dystocia (SD) and arrest of labor due to relative or absolute cephalopelvic disproportion [1, 2]. However, sonography-based EFW is known to have limited reliability with regard to accuracy especially in growth-retarded or macrosomic fetus [3]. The impact of its unrecognized failures may have major effects on mother and child. However, the risk of unnecessary early induction and even more Caesarean section must be carefully weighed against the risk for the child although events like SD are rare and only 10% are associated with sequelae [4].

Data from previous studies on the potential impact of maternal OBS, DM or excess weight gain (EWG) during pregnancy are controversial but suggest that the current method of EFW is prone to significant error. To advise women on the safest mode of delivery on an individual basis, more accurate, real-world data on the expected percent error (%error) based on large and unselected representative populations that take into account the high prevalence of OBS and DM, are necessary. As a consequence, this study aims to 1) evaluate the impact of maternal parameters that are assumed to adversely impact EFW accuracy and the effect of fetal anthropometric parameters and 2) to determine detailed data for the %error that has to be expected in different BW categories.

## Methods

### Design and population

The retrospective data collection includes 19,196 deliveries at three tertiary perinatal centers occurring between 1 January 2014 and 1 January 2017. The centers serve a population of similar ethnic and social background and have similar clinical management standards for women with DM.

The inclusion criteria were singleton pregnancies in women ≥ 18 years old, delivery at ≥ 37 + 0 weeks of gestation and fetal US biometry performed within 14 days to delivery. Gestational age was calculated from the first day of the last menstrual period and was corrected if ultrasound measurements of the crown-rump length during the first trimester deviated > 7 days. Clinical data were obtained from the electronic hospital records and delivery logbooks. Data included maternal age, height, weight and pre-pregnancy body mass index (BMI), gravity, parity, weight gain, obstetrical history and maternal DM status. EWG was defined as exceeding the recommended weight gain ranges given by the Institute of Medicine (IOM) for different maternal BMI groups [5]. Maternal DM status was either pre-existing type I/II or gestational diabetes (GDM), last managed either with dietary or insulin therapy. GDM was diagnosed by a 75 g oral glucose tolerance test performed at 24–27 gestational weeks using the International Association of DM and Pregnancy Study Groups criteria [6]. Neonatal data included gender, length, head circumference (HC) and BW. BW percentiles are based on Voigt et al.’s data published in 2014 [7]. Large for gestational age (LGA) was defined as BW ≥ 90th percentile and small for gestational age (SGA) as ≤ 10th percentile.

## US data

US examinations were performed at visits to the prenatal care clinic or the latest when women present with labor. The majority of the US scans were done by residents above their third year of training. EFW ≤ 10th or ≥ 90th percentile was re-evaluated by fellows or consultants. Ultrasonic devices used: Voluson E8 or E10 (General Electric Healthcare, Milwaukee, WI, USA), Xario SSA-66A (Toshiba) or Acuson X300 (Siemens). Complete US biometry within 14 days of delivery was performed, measuring biparietal diameter (BPD), HC, abdominal circumference (AC) and femur length (FL). Head measurements were obtained in a horizontal section at the level of the thalamus and the cavum septi pellucidi. BPD was measured with the intersection of the calibers placed from the outer edges of the proximal to the distal calvarial wall at the widest part of the skull. The HC was obtained by using an ellipse, which included the outer surface of the cranium. AC measurements were taken at the standard cross-sectional view at the level of the stomach and portal sinus of the liver by placing the line of the ellipse on the outer border of the soft-tissue circumference. The FL was taken when the full femoral diaphysis was seen in a longitudinal section in an image that included the epiphyseal cartilages of the bone. Calipers were placed at each end of the diaphysis. Percentiles of measurements are based on the data of Snijder et al. [8].

EFW were uniformly calculated by the Hadlock IV formula considering HC, AC, BPD and FL [9], EFW percentiles were derived from Hadlock as well. As measures of asymmetrical growth, AC to HC difference and the HC/AC ratio, were calculated. The %error was calculated according to [(EFW − BW))/BW × 100]. A negative mean percentage difference indicates that EFW underestimated the real BW on average and positive values indicate overestimation. The accuracy of the EFW results was further classified as under- or overestimation in the ranges of > 20%, > / ≤ 10%–20% and ≤  ± 10% (accurate) of BW.

## Statistics

Data were organized and analyzed using IBM SPSS Version 25 (SPSS Inc. Chicago, IL, USA). Cases without complete maternal and neonatal parameters were excluded. Descriptive statistics included mean and standard deviation for the continuous normally distributed parameter. Means for continuous variables were compared between cases with EFW > 10% and ≤  ± 10% deviation of BW using Student’s *t*-test for independent sample sizes. Chi-square test was performed to show significance between categorial variables. BW between 2,500 g and 4,500 g was subdivided by increments of 250 g. One-way ANOVA was performed to compare the mean %error of EFW to BW in different BW categories. Forward stepwise logistic regression analysis was performed to evaluate independent predictors for accurate ≤  ± 10% EFW to BW. Presence of OBS (BMI ≥ 30 kg/m^2^), GDM, pre-existing DM, EWG, LGA, BW > 4,000 g, SGA and days of US to delivery > 7–14 were entered as categorical descriptive parameters. All confidence intervals (CI) were 95%. *P* values of < 0.05 were considered significant.

## Results

**Fig. 1:**
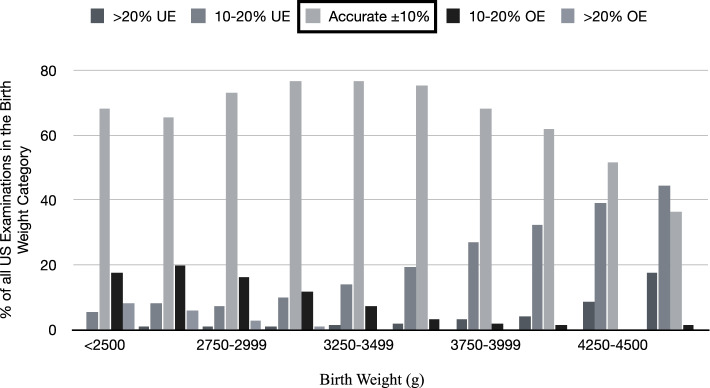
Accuracy of EFW in relation to birth weight. Accurate EFW was assumed when the deviation from birth weight was within ± 10%. UE- Underestimation OE- Overestimation US- Ultrasound

**Fig. 2:**
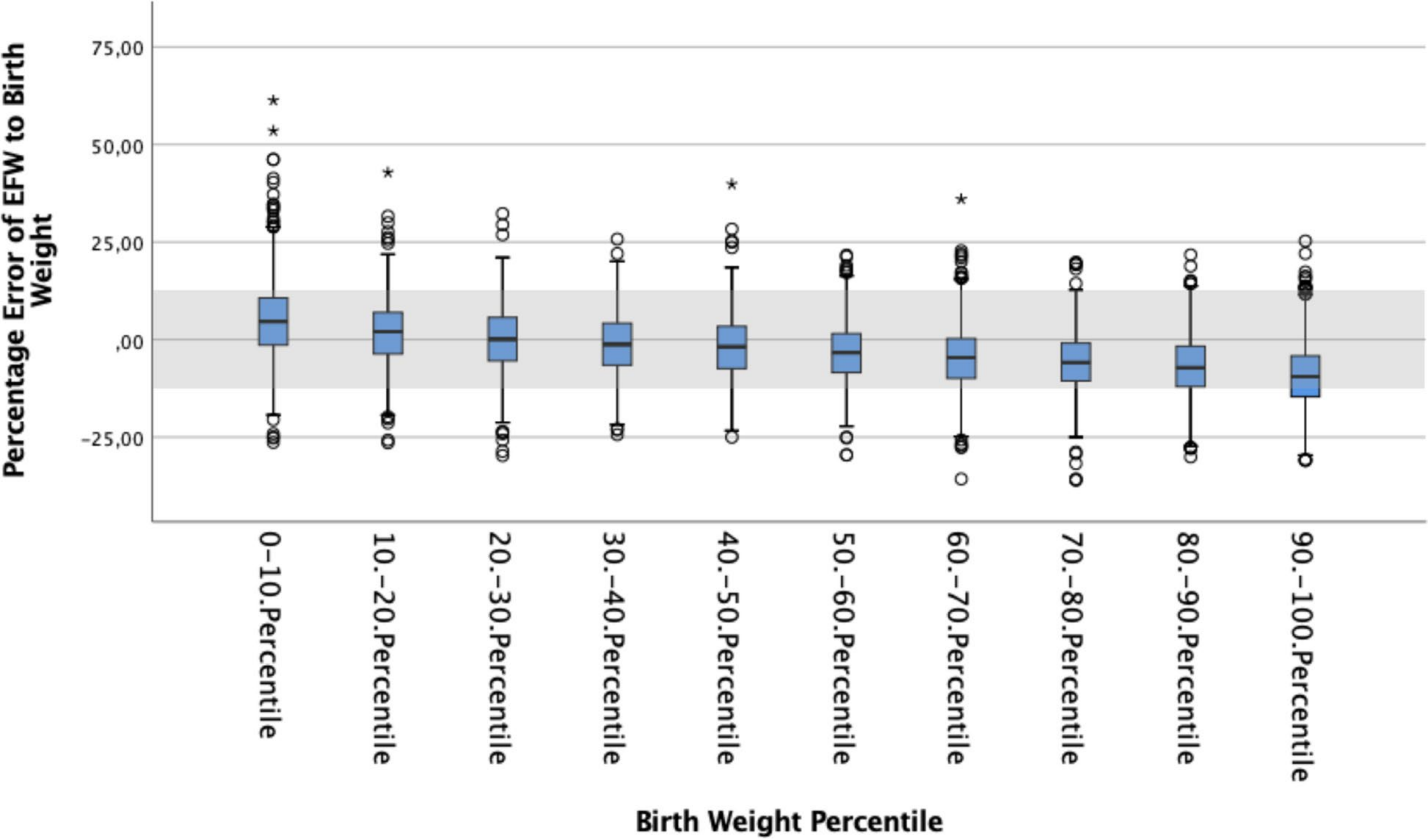
%error according to the birth weight percentiles

**Fig. 3:**
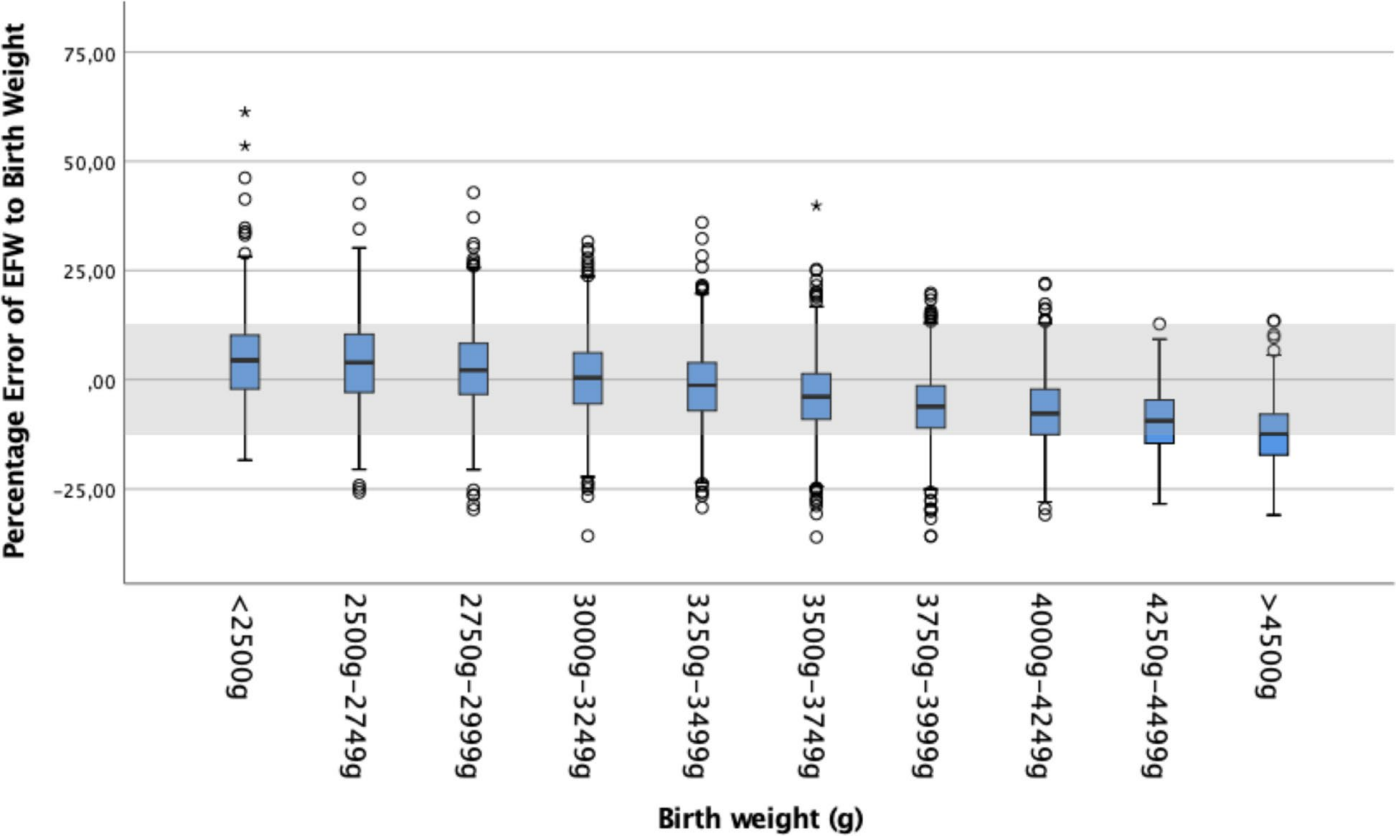
%error according to the birth weight

11,049 cases fulfilled the inclusion criteria and qualified for data analysis. The mean time range between scan and delivery was 4.1 ± 3.9 days. 79.1% (*n* = 8,737) had been performed within 7 days to delivery, partly during admission to the delivery ward. EFW was within 10% of BW in 71.6% (*n* = 7,912). In 2.2% (*n* = 248) and 17.7% (*n* = 1,959), the EFW underestimated the actual BW by > 20% and between 10 and 20%, respectively. An EFW overestimation of > 20% and between 10 and 20% were observed in 1.1% (*n* = 117) and in 7.4% (*n* = 818), respectively. Table 1 summarizes the maternal and neonatal characteristics of the study participants comparing cases with accurate EFW (≤ ± 10%) vs. outlying EFW (> ± 10%). EFW of US examinations performed between 7–14 days to delivery showed a derivation >  ± 10% to BW in 27.7% (*n* = 870) of the cases while the percentage was significantly lower with scans closer to delivery (27.7% vs. 18.2%,* p* < 0.001). In total, 12.5% (*n* = 1,386) of the women were diagnosed with DM, 12.6% (*n* = 1,387) were obese and 44.8% (*n* = 4,945) exceeded the IOM weight gain recommendation. EFW was less frequently accurately predicted in women with pre-existing DM (61.1% vs. 71.7%, crude Odds ratio (cOR) 1.615, confidence interval 95% (CI) 1.126–2.316, *p* = 0.009), while the rate of ≤  ± 10% EFW of BW was not different between pregnancies without or with GDM (71.7% vs. 71.1%, cOR 1.028 CI 0.903–1.179, *p* = 0.678). A tendency of a lower rate of accurately predicted EFW (≤ 10%) was seen with the presence of OBS. However, the difference did not reach significance (69.6% vs. 71.9%, cOR 1.116 CI 0.987- 1.262, *p* = 0.08) and the %error was not significantly different between normal-weight and obese women (− 2.23 ± 9.1 vs. − 2.40 ± 9.38, *p* = 0.18). The mean BMI (kg/m^2^) in women with DM was significantly higher than in women with normal weight (27.2 ± 6.3 vs. 23.86 ± 4.5, *p* < 0.001). 32.1% of the woman diagnosed with DM were obese. Excess of IOM recommendation of weight gain during pregnancy was not associated with a lower rate of accurately predicted EFW (71.2% vs. 72%, cOR 1.040 CI 0.957–1.133, *p* = 0.328). Obese women exceeded the recommendations more frequently than non-obese women (54.7% vs. 43.3%, *p* < 0.001). 12.6% (*n* = 1,394) of the women gave birth to newborns with BW > 4,000 g. In the subpopulation with macrosomia (BW > 4,000 g), maternal DM and OBS occurred in 16.5% (*n* = 230) and 19.1% (*n* = 266), respectively. Macrosomia was documented in pregnancies without maternal DM, with GDM and with pre-existing DM in 12.04%, 15.44% and 26.98%, respectively (*p* < 0.001). Parameters of fetal anthropometrics showed significant differences between cases of EFW within and > 10% (Tab. 1). The distribution of under-, accurate or overestimation of EFW varied with BW (Fig. 1). The highest percentage of accurate EFW was seen with BW between 3,000 g and 3,249 g (79.6%) and decreased to 55.45% (*n* = 773), 44.99% (*n* = 211) and 36.36% (*n* = 76) respectively with BW ≥ 4,000 g, ≥ 4,250 g and ≥ 4,500 g. When BW exceeded 4,000 g, 36.0% and 7.4% of the fetus had been underestimated by 10%–20% and > 20% of BW, respectively, adding up to an underestimation rate of 43.4%. The mean EFW %error from BW was − 2.39% ± 9.13%, (CI − 2.56; − 2.22; min, − 36.03%; max, 61.28%), resulting in an absolute deviation of the maximal under- and overestimation of 1,565 g and 1,394 g, respectively. Table 2 gives the exact numbers of %error for BW categories divided by increments of 250 g. The lowest %error 0.28% ± 8.45% was seen in the group with a BW of 3,000 g–3,249 g. ANOVA showed significant differences of %error for different BW subdivisions (*p* < 0.05). Figure 2 displays the %error according to BW percentiles, while Fig. 3 displays the %error according to the actual BW. Forward stepwise logistic regression analysis evaluated the parameters with the highest influence on US accuracy with EFW deviation of > 10% of BW as the dependent variable. BW > 4,000 g, days between US to delivery > 7–14, LGA and SGA revealed to be significant independent risk factors for EFW >  ± 10% to BW in univariate as well as multivariate regression analysis. (Table 3) The %error for scans performed ≤ 7 days was significantly lower than those performed > 7–14 days (-1.354% vs. -6.344%, *p* < 0.001), revealing an increase in EFW accuracy for scans closer to delivery.

**Table 1:** Maternal and neonatal characteristics with accurate EFW (≤ ± 10%) vs. EFW outlying ± 10%

	EFW ≤ ± 10% *N* = 7912	EFW outlying 10% *N* = 3137	*p*-Value
Maternal characteristics			
Age (Years)	30.8 ± 5.5	30.9 ± 5.6	0.226 ^b^
Parity	1.76 ± 1.1	1.82 ± 1.1	0.737 ^b^
DM	12.3% (973)	13.2% (413)	0.208 ^a^
• GDM (diet./med.)	11.3% (896)	11.6% (1260)	0.678 ^a^
• Pre-existing DM	1.0% (77)	1.6% (49)	0.009 ^a^
Mean pre-pregnancy BMI (kg/m^2^)	24.3 ± 5.0	24.5 ± 5.1	0.136 ^b^
Pre-pregnancy BMI ≥ 30 kg/m^2^	12.4% (966)	13.7% (421)	0.188 ^a^
Weight Gain (kg)	14.3 ± 6.0	14.2 ± 6.2	0.55 ^b^
EWG (IOM)	49.1% (3520)	49.7% (1425)	0.602 ^b^
Days of last US to delivery	3.7 ± 3.7	4.7 ± 4.3	< 0.001 ^b^
Days of last US to delivery > 7–14	18.2% (1439)	27.7% (870)	< 0.001 ^a^
Fetal anthropometrics			
SGA (≤ 10.Perc.)	11.11% (879)	13.42% (421)	0.001 ^a^
LGA (≥ 90. Perc.)	7.18% (568)	17.25% (541)	< 0.001 ^a^
BW ≥ 4000 g	9.4% (743)	19.3% (607)	< 0.001 ^a^
AC Percentile	46.1 ± 24.3	41.7 ± 25.1	0.025 ^b^
AC-HC (cm)	0.69 ± 1.83	0.40 ± 2.0	< 0.001 ^b^
HC/AC-Ratio	0.98 ± 0.05	0.99 ± 0.06	< 0.001 ^b^
Neonatal anthropometrics			
Birth Weight (g)	3416.5 ± 451.6	3522.2 ± 554.9	< 0.001 ^b^
Birth Weight Percentile	46.57 ± 27.69	53.62 ± 32.01	< 0.001 ^b^
Shoulder dystocia (Vaginal delivery mode; *N* = 8,161)	0.7% (44)	1.3% (28)	0.026 ^a^

^a^Chi-square test, ^b^ Student T-test

**Table 2:** Percent error of EFW to actual BW for different categories of BW

Percentage error of EFW to BW			
BW	*N*	Mean ± SD	CI
< 2500 g	278	5.11 ± 10.95	3.81—6.40
2500 g-2749 g	519	3.89 ± 10.27	3.01—4.78
2750 g-2999 g	1054	2.40 ± 9.10	1.85—2.95
3000 g-3249 g	1883	0.28 ± 8.45	-0.10—0.66
3250 g-3499 g	2346	-1.55 ± 8.17	-1.89 -(-1.22)
3500 g-3749 g	2027	-3.83 ± 7.84	-4.17 -(-3.49)
3750 g-3999 g	1548	-6.15 ± 7.56	-6.52 -(-5.77)
4000 g-4249 g	830	-7.28 ± 7.72	-7.80 -(-6.75)
4250 g-4499 g	355	-9.57 ± 7.45	-10.35 -(-8.73)
> 4500 g	209	-12.44 ± 7.97	-13.53 -(-11.36)
*p*-value ^c^	< 0.001		

^c^Oneway ANOVA

**Table 3:** Putative risk factors for EFW >  ± 10% of BW, OR derived from logistic regression analysis

	cOR	CI	aOR	CI	*p*-value
**BMI ≥ 30 kg/m**^**2**^ (12.8%; *N* = 1,389)	1.116	0.987- 1.262	1.002	0.879–1.141	0.978
**GDM** (11.4%; *N *= 1,260)	1.028	0.903–1.170	0.913	0.796–1.048	0.197
**Pre-existing DM** (1.1%; *N *= 126)	1.615	1.126–2.316	1.145	0.785–1.672	0.482
**Days of last US to delivery > 7–14** (20.9%; *N* = 2,309)	1.727	1.567–1.902	1.711	1.549–1.890	< 0.001
**EWG (IOM)** (44.8%; *N *= 4,945)	1.040	0.957–1.133	0.964	0.883–1.052	0.411
**LGA (≥ 90. Perc.)** (10.2%; *N* = 1,125)	2.705	2.386–3.062	2.085	1.729–2.513	< 0.001
**BW ≥ 4000 g** (12.2%; *N* = 1,350)	2.315	2.061–2.601	1.499	1.258–1.786	< 0.001
**SGA (≤ 10.Perc.)** (12.1%; *N* = 1,338)	1.231	1.089–1.392	1.464	1.289–1.664	< 0.001

*cOR*- crude Odds Ratio, *aOR*- adjusted Odds Ratio

## Discussion

In this large-scale dataset of US exams at term comprising a high rate of scans in pregnancies with maternal DM, OBS and EWG as maternal parameters with potential influence on accuracy demonstrated that only OBS had been borderline significantly associated with lower rates of accurate EFW within 10% error to BW. However, the effect diminished after adjustment for fetal growth parameters. BW had a considerable impact on accuracy, with by far the highest %error in newborns with BW > 4,000 g, resulting in 43.4% scans with underestimation. Scans performed < 7 days had markedly lower %error than those obtained 7–14 days before delivery. The exact numbers for %error for BW categories divided by 250 g were calculated to specify the extent of EFW deviation from actual BW.

In contrast to common clinical assumptions, the data in this study indicate that the presence of DM did not diminish EFW accuracy except for pre-existing DM. Prior studies report either a slight but insignificant difference in EFW deviation from BW [10], greater actual amount of deviation but the same %error [11] or lower accurate prediction rates only in women with pre-existing DM [12]. Poorly controlled DM, even with moderate hyperglycemia, increases the rate of accelerated growth [13]. In the population of this study, macrosomia was more frequent in women with DM, and %error increased with increasing BW. The BW rate of > 4,000 g was almost twice as high with pre-existing DM compared to GDM (26.9% vs. 15.4%), which is likely the reason for lower EFW accuracy with pre-existing DM. Thus, the impact of DM on US accuracy is likely to be mediated by frequent excess fetal growth, the OR was no longer significant when adjusted for fetal growth.

Another aspect that may create greater concern of accuracy of EFW in women with DM is the frequent co-incidence of OBS with GDM and type II DM as seen in the current population. Mothers with DM had significantly higher BMI. OBS was associated with fewer scans with accurate predicted BW, however as seen for DM, the OR was no longer significant when adjusted for fetal growth. Thus, neither DM, nor OBS is a risk factor by it’s own for fetal weight estimates >  ± 10% of actual BW. The published data regarding OBS are as conflicting as in DM. Some studies similarly demonstrated a lower EFW accuracy with increasing maternal BMI. However, limited numbers involving less than one-tenth of the cases that this study incorporated were assessed [14, 15]. In contrast, Gonzalez et al. suggest no influence of BMI on EFW accuracy in data collected from 403 pregnancies [16]. Non-differentiation of subgroups distinguishing overweight from OBS may be a reason for contrary findings.

BW deviation was the major factor that reduced the accuracy of EFW near term, more evident in macrosomic than in growth-retarded fetus. Prior studies confirm that SGA fetus tend to be overestimated [17], while the LGA fetus had been underestimated [18]. Measurements may be unconsciously influenced by the sonographer’s desire to avoid clinical consequences of the diagnosis of severe growth deviation. Besides this potential psychological factor, the precise demarcation of the abdomen becomes more difficult with increasing size. The underestimation of high fetal weight may result in a higher risk of SD and arrest of labor due to cephalopelvic disproportion.

The %error of EFW is in part also a result of the calculation by an empiric formula and not only of the difficulties to measure accurately head, abdomen and femur, the three defined sites of the fetus that are entered in the formulas. Numerous different formulas had been developed over the last decades with more or less the same problems to predict BW [19].

One formula specified for estimates > 4,000 g, including maternal weight in the calculation provided a higher rate of estimates within 10% of BW and lower %error [20]. Another confounder, the examiner-dependent parameter, can be seen as a limitation of this study because the impact of varying experiences of the examining physicians in this study cannot be quantified. Stubert et al. concluded that consultants exhibit a significantly higher EFW accuracy rate than residents especially in the group of newborns with a birth weight of ≥ 4,000 g [21]. However, in our study EFW in the upper and lower ranges had been routinely re-evaluated by consultants. Another limitation is the possible non-transferability of the study’s findings to suburban clinics because higher expertise and higher equipment standard in tertiary perinatal centers can be assumed. The three centers serve as specialized obstetrical units for the care of pregnant women with DM, resulting in potentially lower macrosomia rate and weight gain. But the high level of medical care with a large percentage of women with DM and/or OBS can be also considered as a study’s strength.

EFW often guides the counselling regarding the mode of delivery and/or need for induction. The sonographic EFW inaccuracy in fetal macrosomia appears to have a greater impact on the mode of delivery than the BW itself. In a prior study, the caesarean section rate was significantly higher (28.48%) with false-positive estimates than with false-negative estimates [22]. Overall, the current accuracy of EFW formulae with conventional biometric parameters by 2D ultrasound seems to have reached its limits. Taking into account that the known limits of fetal weight estimation by US need to be dealt with for the moment, it is clinically relevant to be able to estimate the specific extent of %error that is to be expected. Therefore, the data in this study was used to calculate the %error for 10 categories of BW including 1,394 cases with BW of ≥ 4,000 g. In addition, the %error was provided according to BW percentiles.

In conclusion, this study examines the influence of both maternal and neonatal parameters on EFW accuracy at term in a population with high rates of DM and OBS. The data of this study disprove the clinical concern that fetal weight estimation is less precise in pregnancies with DM per se. Even OBS had a limited impact. Less accuracy is mediated by high BW, which often goes along with DM and/or OBS. BW deviation especially in the upper range has by far the highest impact on EFW accuracy. Therefore, data determining the extent of %error for different BW categories were provided to allow delivery planning involving the given antenatal range of potential EFW deviation to actual BW.

## Data Availability

The datasets used and/or analyzed during the current study are available from the corresponding author on reasonable request.

## Abbreviations

US: Ultrasonography
EFW: Estimation of fetal weight
BW: Birth weight
DM: Diabetes
OBS: Obesity
SD: Shoulder dystocia
EWG: Excess weight gain
%error: Percent error
BMI: Body mass index
IOM: Institute of Medicine
GDM: Gestational diabetes
HC: Head circumference
LGA: Large for gestational age
SGA: Small for gestational age
BPD: Biparietal diameter
AC: Abdominal circumference
FL: Femur length
ANOVA: Analysis of variance
CI: Confidence interval 95%
cOR: Crude odds ratio
aOR: Adjusted odds ratio
